# PBPK modeling of the antidepressant doxepin incorporating CYP2D6 genotype for precision pharmacotherapy

**DOI:** 10.1007/s12272-026-01600-5

**Published:** 2026-02-27

**Authors:** Joohee Seok, Nahyun Kang, Je Jin Lee, Chang-Keun Cho, Yun Jeong Lee

**Affiliations:** 1 Chung-Ang University College of Medicine, Seoul, 06974 Korea; 2 School of Pharmacy, Sungkyunkwan University, Suwon, 16419 Republic of Korea; 3 Department of Pharmaceutics, Center for Pharmacometrics and Systems Pharmacology, College of Pharmacy, University of Florida, Orlando, FL 32827 USA; 4 College of Pharmacy, Dankook University, Cheonan, 31116 Republic of Korea

**Keywords:** Doxepin, *N*-desmethyldoxepin, CYP2D6, Pharmacogenomics, Physiologically based pharmacokinetic modeling, Precision pharmacotherapy

## Abstract

Doxepin, a tricyclic antidepressant, exhibits substantial interindividual variability in pharmacokinetics, mainly attributable to genetic polymorphisms of cytochrome P450 (CYP) enzymes, particularly CYP2D6. Such variability may lead to clinically relevant differences in drug exposure, therapeutic response, and adverse effects when doxepin is used for antidepressant therapy. The present study aimed to develop and validate physiologically based pharmacokinetic (PBPK) models of doxepin and its active metabolite, *N*-desmethyldoxepin, incorporating CYP2D6 genetic polymorphisms to support precision pharmacotherapy. PBPK models were constructed using PK-Sim^®^ (version 12.0) based on published clinical pharmacokinetic and pharmacogenomic data obtained after oral administration of doxepin. Model development was performed in a non-genotyped population. Subsequently, it extended to CYP2D6 ultra-rapid (UM), normal (NM), intermediate (IM), and poor metabolizer (PM) phenotypes by integrating genotype-specific metabolic activity. Model performance was evaluated by comparing predicted plasma concentration–time profiles, area under the concentration–time curve (AUC), and maximum plasma concentration (C_max_) with observed clinical data. The model's predictive capability was further assessed using goodness-of-fit analysis, visual predictive checks, and geometric mean fold error (GMFE). The PBPK models adequately reproduced the observed pharmacokinetics of doxepin and *N*-desmethyldoxepin, with most predicted AUC and C_max_ values falling within a twofold error range across both non-genotyped and CYP2D6-genotyped populations. Simulations demonstrated an apparent genotype-dependent increase in doxepin exposure as CYP2D6 metabolic capacity decreased, consistent with clinical observations. Similarly, *N*-desmethyldoxepin exposure showed an inverse relationship with CYP2D6 activity, reflecting differences in metabolite formation and elimination. Despite the limited availability of genotype-specific concentration–time data for the metabolite, the model captured observed exposure trends, supporting its mechanistic plausibility. In conclusion, a PBPK framework integrating CYP2D6 genetic polymorphisms was successfully developed for doxepin and its active metabolite. The model reliably predicts genotype-dependent pharmacokinetic variability and provides a mechanistic basis for individualized dose optimization. This PBPK approach may serve as a valuable tool to support precision pharmacotherapy for doxepin, particularly in the context of antidepressant treatment.

## Introduction

Doxepin, a tricyclic antidepressant (TCA), has been prescribed for the treatment of major depressive disorder, anxiety disorders, and insomnia (Richelson et al., 1984; Shimamura et al. 2011). Although doxepin is not considered a first-line antidepressant due to its unfavorable adverse-effect profile compared with newer antidepressants, it may still be helpful in selected patients with depression accompanied by prominent anxiety or sleep disturbances (Pinder et al. 1977).

Doxepin is extensively metabolized in the liver, primarily by cytochrome P450 (CYP) enzymes. CYP2C19 catalyzes the *N*-demethylation of doxepin to its pharmacologically active metabolite, *N*-desmethyldoxepin (nordoxepin), whereas CYP2D6 is mainly responsible for the further metabolism of both doxepin and *N*-desmethyldoxepin to inactive metabolites (Haritos et al. 2000; Härtter et al. 2002; Kirchheiner et al. 2005).

Doxepin undergoes extensive hepatic metabolism, with less than 3% of the administered dose excreted unchanged in the urine. The drug is administered as a mixture of approximately 85% *E*-(trans)-isomer and 15% *Z*-(cis)-isomer (Bogaert et al. 1981; Hrdina et al. 1990; Midha et al. 1992; Adamczyk et al. 1995; Deuschle et al. 1997). Although some differences in pharmacological activity between the two isomers have been reported (Kaneko et al. 2024), no clinically meaningful differences in absorption or metabolic rates have been observed. Consequently, the pharmacokinetics of doxepin in patients, as well as therapeutic drug monitoring (TDM), are generally based on measurements of the total isomeric mixture rather than individual stereoisomers (Hrdina et al. 1990).

The major metabolite, *N*-desmethyldoxepin, exhibits antidepressant efficacy comparable to that of the parent compound, while it shows less pronounced sedative effects (Dawkins et al. 1994). Accordingly, the clinical antidepressant response to doxepin correlates more strongly with the combined plasma concentrations of doxepin and *N*-desmethyldoxepin than with doxepin alone (Ward et al. 1982; Faulkner et al. 1983; Leucht et al. 2001). In contrast, the hypnotic effect of doxepin is primarily mediated by the parent compound.

Importantly, doxepin is used at markedly different doses and via distinct pharmacological mechanisms depending on the therapeutic indication. For the treatment of depression and anxiety, therapy typically begins at low doses (e.g., 25 mg/day), followed by gradual titration at intervals of 5–7 days up to a maximum single daily dose of approximately 150 mg (FDA 2007, 2014). In contrast, for the treatment of insomnia, ultra-low doses of 3–6 mg are recommended, administered approximately 30 min before bedtime (Schroeck et al. 2016; Matheson and Hainer 2017). The antidepressant and anxiolytic effects are primarily mediated through inhibition of norepinephrine and serotonin reuptake at presynaptic nerve terminals, resulting in increased synaptic neurotransmitter concentrations (Katwala et al. 2013; Yeung et al. 2015; Almasi et al. 2024). Conversely, the hypnotic effect of low-dose doxepin is mainly attributable to selective antagonism of histamine H_1_ receptors in the cerebral cortex (Singh and Becker 2007; Katwala 2013; Yeung et al. 2015; Schroeck et al. 2016; Matheson and Hainer 2017).

Both plasma concentrations and clinical responses to doxepin exhibit substantial interindividual variability (Ereshefsky et al. 1988; Hrdina et al. 1991; Kirchheiner et al. 2005; Funk et al. 2022). This variability arises from a combination of factors, including genetic polymorphisms in drug-metabolizing enzymes, patient physiological characteristics, and drug–drug interactions. As noted above, plasma concentrations of doxepin and its active metabolite *N*-desmethyldoxepin are significantly influenced by CYP2C19 and CYP2D6 genotypes. However, the clinical implications of this variability differ markedly depending on the therapeutic context (Asnis et al. 2015). At antidepressant doses, variability in metabolism leads to clinically relevant differences in both efficacy and toxicity due to a steep concentration–response relationship (Burke and Preskorn 1999; Baumann et al. 2004; Funk et al. 2022). In contrast, at the ultra-low doses used for insomnia, the pharmacodynamic effect is primarily driven by H_1_ receptor antagonism at concentrations far below those associated with tricyclic antidepressant activity, rendering interindividual variability primarily clinically benign (Roth et al. 2007; Katwala et al. 2013; Rojas-Fernandez 2014; Asnis et al. 2015).

Furthermore, because the antidepressant effect of doxepin depends on combined exposure to doxepin and *N*-desmethyldoxepin, variability in CYP2C19 genotype is generally less clinically significant (Kleine Schaars et al. 2026). The Royal Dutch Pharmacists Association-Pharmacogenetics Working Group concluded that therapy adjustment is not required for doxepin and CYP2D19 (Kleine Schaars et al. 2026). In contrast, the CYP2D6 genotype has a pronounced impact on total active exposure, and consideration of the CYP2D6 polymorphism is therefore recommended when doxepin is used for antidepressant therapy (Hicks et al. 2013, 2017; Kleine Schaars et al. 2026).

CYP2D6 exhibits extensive genetic polymorphism, with more than 185 allelic variants identified (https://www.pharmvar.org/gene/cyp2d6, accessed on Jan 10, 2026). These polymorphisms lead to vast interindividual differences in enzyme activity and, consequently, in drug exposure (Bertilsson et al. 2002; Preskorn et al. 2009; Ning et al. 2018; Byeon et al. 2019). CYP2D6 phenotypes are typically categorized as ultra-rapid (UM), normal (NM), intermediate (IM), or poor metabolizers (PM), and the distribution of these phenotypes varies by ethnicity (Griese et al. 1999; Wan et al. 2001; Qin et al. 2008; de Leon et al. 2009; Hosono et al. 2009; Pietarinen et al. 2016; Byeon et al., 2018a; Bae et al. 2020). Pharmacokinetic studies demonstrated that CYP2D6 PMs have significantly higher plasma concentrations of doxepin and *N*-desmethyldoxepin than NMs or UMs, thereby increasing the risk of adverse effects (Kirchheiner et al. 2005). Conversely, UMs may exhibit subtherapeutic plasma levels, predisposing them to treatment failure.

Several strategies have been proposed to achieve precision pharmacotherapy; among them, physiologically based pharmacokinetic (PBPK) modeling that incorporates pharmacogenetic information has emerged as one of the most effective approaches (Cho et al. 2023, 2024a, 2024b, 2024c; Park et al. 2025). Accordingly, the present study aimed to develop a PBPK model of doxepin that integrates CYP2D6 genotype to support individualized dose optimization for precision pharmacotherapy in antidepressant treatment.

## Materials and methods

### Software

PBPK models of doxepin and its active metabolite, *N*-desmethyldoxepin, were developed using PK-Sim^®^ version 12.0 (Bayer AG, Leverkusen, Germany). The software was also used for parameter identification and sensitivity analyses. Plasma concentration–time profiles available only as graphical outputs in previously published studies were digitized using Engauge Digitizer^®^ version 12.1 (https://markummitchell.github.io/engauge-digitizer/), following the standardized digitization workflow proposed by Wojtyniak et al. (2020).

### Clinical pharmacokinetic data

Published clinical pharmacokinetic (PK) studies of orally administered doxepin were systematically reviewed. Data from film-coated tablets administered to fasting healthy adults were extracted, including demographic characteristics, genotypes, dosing regimens, PK parameters, and plasma concentration–time profiles. Studies were grouped into non-genotyped and genotyped populations (*CYP2D6* UM, NM, IM, and PM). Datasets were divided into model development and validation sets to ensure independence during performance evaluation.

### Model workflow

PBPK models of doxepin and its active metabolite were built based on a "middle-out" strategy. First, PBPK models for doxepin and *N*-desmethyldoxepin were built in non-genotyped populations (Hanke et al. 2020). Next, the established PBPK model in those populations was scaled to populations with different *CYP2D6* genotypes. Model input parameters were refined by iteratively performing sensitivity analyses and parameter identification based on the observed data at each step.

### Model Building

Previously published studies and drug databases were reviewed to collect physicochemical and ADME (absorption, distribution, metabolism, and excretion) properties of doxepin and its active metabolite. The collected data were incorporated into the PBPK model. Most physicochemical parameters were used as reported, except for the Log P of doxepin, which was slightly reduced from 3.84 to 3.60 to improve model fitting. Because no reported Log P value was available for *N*-desmethyldoxepin, this parameter was optimized during model development.

Species-specific physiological parameters and quantitative structure–activity relationship (QSAR) data included in the PK-Sim^®^ software were used and optimized as appropriate. The metabolic pathway describing the CYP2C19-mediated demethylation of doxepin to *N*-desmethyldoxepin was implemented in the PBPK model, followed by inclusion of CYP2D6-mediated hydroxylation of both doxepin and *N*-desmethyldoxepin.

For the doxepin model, the Michaelis–Menten constant (K_m_) for CYP2D6 was adopted from Haritos et al. (2000), whereas the K_m_ value for CYP2C19 and the turnover numbers (k_cat_) for both CYP2D6 and CYP2C19 were optimized to reproduce the plasma concentration–time profiles of doxepin and its metabolite in a non-genotyped population. Subsequently, k_cat_ values were further refined according to *CYP2D6* genotype. The reference enzyme concentrations for CYP2D6 and CYP2C19 (0.40 μmol/L and 0.76 μmol/L, respectively) were adopted from the default PK-Sim^®^ database (Rodrigues 1999).

In the *N*-desmethyldoxepin model, parameters associated with CYP2D6 metabolism were optimized separately, and total hepatic clearance was introduced to represent the remaining metabolic pathways of *N*-desmethyldoxepin. This parameter was optimized accordingly.

Renal clearance was excluded from the model because less than 3% of doxepin is excreted unchanged or as nordoxepin in urine, rendering renal elimination negligible (Yan et al. 2002).

Doxepin tablet dissolution was described using a Weibull function, and relevant parameters were adjusted to capture both the time to maximum plasma concentration (T_max_) and the initial absorption delay (50% dissolution time: 12.0 min; shape factor: 0.80). Dosing regimens for each simulation were aligned with the corresponding clinical study designs. Tissue-to-plasma partition coefficients were estimated using the Rodgers and Rowland method (Rodgers and Rowland 2006) for doxepin and the Schmitt method (Willmann 2007; Loizou 2008) for *N*-desmethyldoxepin. Cellular permeability values for both compounds were predicted using the standard PK-Sim^®^ methods (Hindmarsh et al. 2025). Model parameters were optimized using the Levenberg–Marquardt algorithm within the parameter identification tool. Dissolution data for doxepin were obtained from Schioppi et al. (2018).

### Sensitivity analysis

A sensitivity analysis was conducted to identify the input parameters that most strongly influenced the area under the plasma concentration–time curve from time zero to infinity (AUC_inf_) and maximum plasma concentration (C_max_) of doxepin and *N*-desmethyldoxepin. Parameters closely related to the optimized model or to the computational procedures implemented in the model were included in the analysis. Sensitivity values were calculated according to the following equation (Eq. 1).

1 $$S=\frac{\Delta PK}{PK}\div \frac{\Delta p}{p}$$

where $S$ is the sensitivity, $PK$ is the initial value of the pharmacokinetic parameter, $\Delta PK$ is the change from the initial value of the pharmacokinetic parameter, $p$ is the initial value of the examined parameter, and $\Delta p$ is the change from the initial value of the examined parameter, respectively. A sensitivity of + 1.0 indicates that a + 10% change in the examined input parameter would result in a + 10% change in the predicted pharmacokinetic parameters.

### Model evaluation

Both graphical and numerical approaches were employed to evaluate the PBPK model. During model development, the predicted plasma concentration–time profiles were visually compared with the observed data by plotting the arithmetic mean and the 90% prediction intervals (i.e., the 5th and 95th percentiles) for a comparable virtual population (n = 100) generated based on the demographic characteristics of subjects in each clinical study. When demographic data were unavailable in the literature, they were randomly generated using the algorithm implemented in PK-Sim^®^ software.

For quantitative evaluation, a twofold error range for AUC and C_max_ was adopted as the acceptance criterion. The fold error value was calculated according to Eq. 2.

2 $$Fold error=\frac{Predicted value}{Observed value}$$

Predicted plasma concentration–time profiles of doxepin and *N*-desmethyldoxepin were compared with the corresponding observed data. Goodness-of-fit plots were generated to evaluate the deviation between predicted and observed plasma concentrations, as well as the AUC and C_max_ values for each profile. Model predictive performance was further quantitatively assessed by calculating the geometric mean fold error (GMFE) for predicted AUC and C_max_ values, as described in Eq. 3.

3 $$GMFE={10}^{x}, with x=\frac{1}{n}\sum_{i=1}^{n}\left|{log}_{10}(\frac{{PK}_{pred,i}}{{PK}_{obs,i}})\right|$$

where ${\mathrm{PK}}_{\mathrm{pred},\mathrm{i}}$ is the i-th predicted AUC or C_max_ value, ${\mathrm{PK}}_{\mathrm{obs},\mathrm{i}}$ is the corresponding observed value, and n is the number of the collected pharmacokinetic parameter data.

## Results

### Model building and evaluation

Clinical pharmacokinetic data for doxepin and *N*-desmethyldoxepin obtained after a single oral dose of 75 mg doxepin were used to develop the PBPK model in the non-genotyped population. In addition, CYP2D6 phenotype–specific pharmacogenomic datasets (UM, NM, IM, and PM) were used to extend and evaluate genotype-specific models. Detailed demographic, genotype, and dosing information for the datasets used in the non-genotyped and genotyped models is summarized in Tables 1 and 2, respectively. Summaries of the input parameters for the doxepin and *N*-desmethyldoxepin PBPK models are provided in Tables 2 and 3, respectively.

**Table 1:** Demographic and dose characteristics of clinical studies used to develop PBPK models of doxepin and *N*-desmethyldoxepin in CYP2D6 genotyped and non-genotyped populations

References		*CYP2D6*	n	Demographic data		
	Dose			Female (%)	Age (year)	Weight (kg)
Doxepin						
Geister et al. (2001)^a^	75 mg Capsule^b^ S.D.	–	30	63	22–50	55–90
Kirchheiner et al. (2005)	75 mg Tablet S.D.	NM	19	0	23–35	50–93
Kirchheiner et al. (2005)	75 mg Tablet S.D.	IM	8	0	26–42	63.0–87.4
Kirchheiner et al. (2005)	75 mg Tablet S.D.	PM	11	0	28–56	69.4–86.2
Kirchheiner et al. (2005)	75 mg Tablet S.D.	UM	11	0	22–44	65.9–92.1
*N*-desmethyldoxepin						
Geister et al. (2001)^a^	75 mg Capsule^b^ S.D.	–	30	63	22–50	55–90
Kirchheiner et al. (2005)	75 mg Tablet S.D.	NM	19	0	23–35	50–93
Kirchheiner et al. (2005)	75 mg Tablet S.D.	IM	8	0	26–42	63–87.4
Kirchheiner et al. (2005)	75 mg Tablet S.D.	PM	11	0	32–60	69.4–86.2
Kirchheiner et al. (2005)	75 mg Tablet S.D.	UM	11	0	22–44	65.9–92.1

^a^Data used for the model development,

^b^The 75 mg capsule dose was administered as three 25 mg immediate-release capsules

Age and weight data are expressed as the range (min–max)

*S.D.* single dose, *n* number of subjects,

*NM* normal metabolizer, *IM* intermediate metabolizer, *PM* poor metabolizer, *UM* ultra-rapid metabolizer

**Table 2:** Summary input parameters for the development of doxepin PBPK model

Parameter (unit)	Reference value	Input value	References/Comments
Basic physico-chemistry			
Molecular weight (g/mol)	279.37	279.37	Dell'Aquila (2002)
Log P	3.84–4.29	3.70	DrugBank, Optimized by PK-Sim^®^
Compound type	–	Basic compound (lipophilic amine, TCA)	–
pK_a_	9.76	9.76 (Basic)	Thiebault et al. (2015), DrugBank
f_u_ (%)	15.9–21.4	20	Virtanen et al. (1982)
Solubility (mg/L)	31.60(pH 7)	31.60(pH 7)	Yalkowsky et al. (2010)
Absorption			
Specific intestinal permeability (cm/min)	–	7.86×10^–4^	Calculated by PK-Sim^®^
Distribution			
Specific organ permeability (cm/min)	–	0.30	Calculated by PK-Sim^®^
Metabolism			
CYP2C19 K_m_ (μM)	–	5	Optimized by PK-Sim^®^
CYP2C19 k_cat_ (1/min)	–	28	Optimized by PK-Sim^®^
CYP2D6 K_m_ (μM)^a^	8	8	Haritos et al. (2000)
CYP2D6 k_cat_ (1/min)	–	260	Optimized by PK-Sim^®^
CYP2D6 k_cat_ (1/min), UM	–	505	Optimized by PK-Sim^®^
CYP2D6 k_cat_ (1/min), NM	–	299	Optimized by PK-Sim^®^
CYP2D6 k_cat_ (1/min), IM	–	85	Optimized by PK-Sim^®^
CYP2D6 k_cat_ (1/min), PM	–	0	Optimized by PK-Sim^®^
Formulation			
Dissolution time (min)	–	12	Schioppi et al. (2018)
Dissolution shape	0.75 < *b* < 1	0.80	Costa et al. (2001), Papadopoulou et al. (2006)

^a^The same K_m_ value was applied across CYP2D6 genotypes; activity differences were reflected via enzyme abundance or Cl_int_

*Log P* logarithm of octanol/water partition coefficient, *pK*_*a*_ negative logarithm of acid dissociation constant, *f*_*u*_ fraction unbound in plasma, *K*_*m*_ Michaelis–Menten constant, *k*_*cat*_ turnover number*, NM* normal metabolizer, *IM* intermediate metabolizer, *PM* poor metabolizer, *UM* ultrarapid metabolizer, *b* dissolution shape

**Table 3:** Summary input parameters for the development of *N*-desmethyldoxepin PBPK model

Parameter (unit)	Reference value	Input value	References/comments
Basic physico-chemistry			
Molecular weight (g/mol)	265.35	265.35	Drug Bank
Log P		3.60	Optimized by PK-Sim^®^
pK_a_	10.47	10.47	DrugBank
f_u_ (%)	–	21	Optimized by PK-Sim^®^
Solubility (mg/L)	0.00361	0.00361	Drug Bank
Absorption			
Specific intestinal permeability (cm/min)	–	7.87×10^–4^	Calculated by PK-Sim^®^
Distribution			
Specific organ permeability (cm/min)	–	0.33	Calculated by PK-Sim^®^
Metabolism			
CYP2D6 K_m_ (μM)	–	40	Optimized by PK-Sim^®^
CYP2D6 k_cat_ (1/min)	–	160	Optimized by PK-Sim^®^
CYP2D6 k_cat_ (1/min), UM	–	505	Optimized by PK-Sim^®^
CYP2D6 k_cat_ (1/min), NM	–	165	Optimized by PK-Sim^®^
CYP2D6 k_cat_ (1/min), IM	–	30	Optimized by PK-Sim^®^
CYP2D6 k_cat_ (1/min), PM	–	0	Optimized by PK-Sim^®^
Total hepatic clearance (L/min)	–	2.50	Optimized by PK-Sim^®^

*Log P* logarithm of octanol/water partition coefficient, *pK*_*a*_ negative logarithm of acid dissociation constant, *f*_*u*_ fraction unbound in plasma, *K*_*m*_ Michaelis–Menten constant, *k*_*cat*_ turnover number, *NM* normal metabolizer, *IM* intermediate metabolizer, *PM* poor metabolizer, *UM* ultrarapid metabolizer

**Fig. 1:**
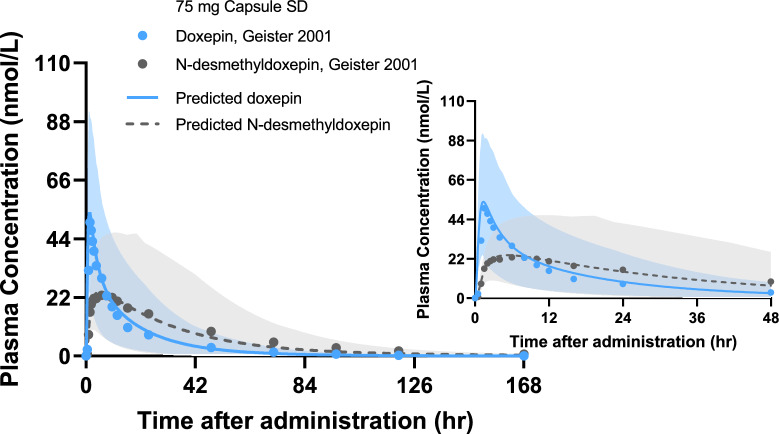
Predicted and observed plasma concentration–time profiles of doxepin and *N*-desmethyldoxepin following a single oral dose of doxepin in non-genotyped population (development dataset). Solid lines indicate the arithmetic mean of predicted plasma concentrations and shaded areas indicate the 5^th^ and 95^th^ percentiles of predicted plasma concentrations. SD: single dose

**Fig. 2:**
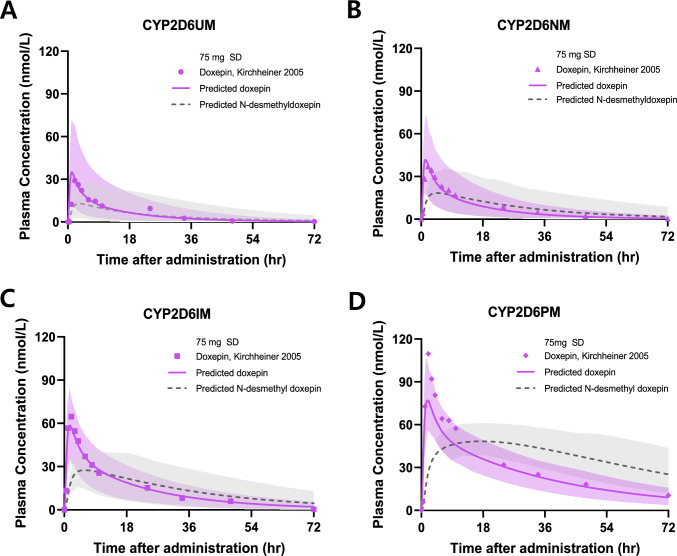
Predicted and observed plasma concentration–time profiles of doxepin and *N*-desmethyldoxepin following a single oral dose of doxepin in *CYP2D6*-genotyped populations. Panels represent **A** ultrarapid metabolizers (UM), **B** normal metabolizers (NM), **C** intermediate metabolizers (IM), and **D** poor metabolizers (PM). Solid lines indicate the arithmetic mean of predicted plasma concentrations and shaded areas indicate the 5^th^ and 95^th^ percentiles of predicted plasma concentrations. SD: single dose

In the non-genotyped model, the predicted plasma concentration-time profiles of doxepin and its active metabolite showed good agreement with the observed data (Fig. 1). Most observed plasma concentrations were contained within the 90% prediction intervals. The goodness-of-fit plot for the non-genotyped model is presented in Fig. 2, and detailed quantitative results are summarized in Table 4. For the non-genotyped population, the fold error values for AUC ranged from 0.85 to 1.02. In contrast, those for C_max_ ranged from 0.98 to 1.02, indicating that all predictions were within the predefined twofold acceptance criterion. In the non-genotyped population, GMFE values for AUC_inf_ and C_max_ ranged from 1.06 to 1.21 for doxepin and from 1.13 to 1.14 for *N*-desmethyldoxepin. In genotype-specific evaluations, the corresponding GMFE values ranged from 1.13 to 1.22 for doxepin and from 1.19 to 1.61 for *N*-desmethyldoxepin, reflecting acceptable predictive performance across CYP2D6 phenotypes.

**Table 4:** Comparison between predicted and observed AUC, C_max_ and T_max_ of doxepin and *N*-desmethyldoxepin after a single oral dose of doxepin across non-genotyped populations and *CYP2D6* genotypes

References	Dose	*CYP2D6*	AUC (nmol·hr/L)^a^			C_max_ (nmol/L)^b^			T_max_ (h)^c^		
			Observed	Predicted	Fold error	Observed	Predicted	Fold error	Observed	Predicted	Fold error
Doxepin											
Geister et al. (2001)^d^	75 mg Capsule^e^ S.D.	–	692.47	709.02	1.02	57.13	55.85	0.98	1.98	1.40	0.71
Kirchheiner et al. (2005)	75 mg Tablet S.D.	NM	398.00	504.66	1.27	35.00	43.97	1.26	-	-	-
Kirchheiner et al. (2005)	75 mg Tablet S.D.	IM	933.00	1,031.62	1.11	62.00	60.26	0.97	-	-	-
Kirchheiner et al. (2005)	75 mg Tablet S.D.	PM	2,291.00	2,315.53	1.01	100.00	78.72	0.79	-	-	-
Kirchheiner et al. (2005)	75 mg Tablet S.D.	UM	331.00	379.95	1.15	26.00	36.19	1.39	-	-	-
*N*-desmethyldoxepin											
Geister et al. (2001)^d^	75 mg Capsule^e^ S.D.	–	1,121.40	955.80	0.85	24.64	25.10	1.02	4.52	5.55	1.23
Kirchheiner et al. (2005)	75 mg Tablet S.D.	NM	562.00	628.00	1.12	25.00	19.14	0.77	–	–	–
Kirchheiner et al. (2005)	75 mg Tablet S.D.	IM	912.00	1,194.33	1.31	28.00	28.42	1.02	–	–	–
Kirchheiner et al. (2005)	75 mg Tablet S.D.	PM	1,820.00	4,640.32	2.55	42.00	49.53	1.18	–	–	–
Kirchheiner et al. (2005)	75 mg Tablet S.D.	UM	200.00	342.48	1.71	16.00	13.42	0.84	–	–	–

Observed and predicted data are given as the mean

^a^*AUC* area under the plasma concentration–time curve from 0 to infinity,^b^C_max_ maximum plasma concentration, ^c^T_max_ time to reach C_max_

^d^Data used for the model development, ^e^The 75 mg capsule dose was administered as three 25 mg immediate-release capsules

*S.D.* single dose, *NM normal* metabolizer, *IM* intermediate metabolizer, *PM* poor metabolizer, *UM* ultra-rapid metabolizer

**Fig. 3:**
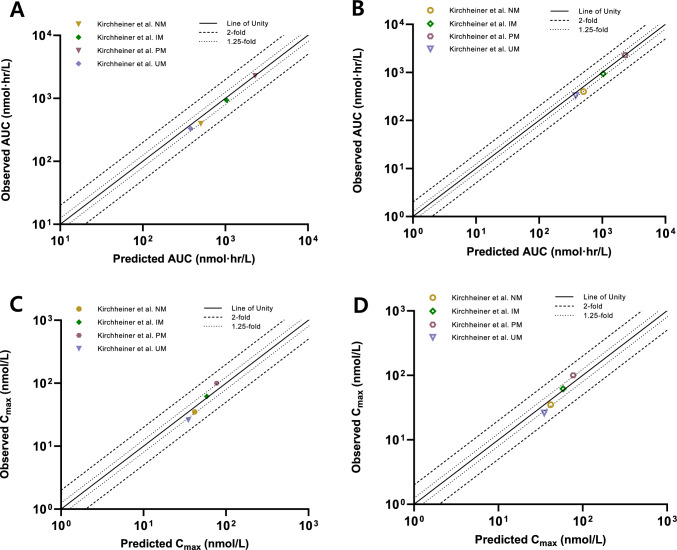
Goodness-of-fit plots comparing the predicted and observed values for AUC of doxepin (**A**) and *N*-desmethyldoxepin (**B**), C_max_ of doxepin (**C**) and *N*-desmethyldoxepin (**D**). Closed symbols represent doxepin, and open symbols indicate *N*-desmethyldoxepin. Circle, diamond, hexagon, and inverted triangle symbols indicate CYP2D6 NM, IM, PM and UM phenotype, respectively. Solid lines indicate the line of unity, dashed lines indicate the twofold range, and dotted lines indicate the 1.25-fold range. AUC: area under the plasma concentration–time curve, C_max:_ maximum plasma concentration.

The predicted plasma concentration–time profiles of doxepin showed good visual agreement with the observed data across all *CYP2D6* genotypes (Fig. 2). Most observed concentrations fell within the 90% prediction intervals. Goodness-of-fit plots comparing the predicted and observed AUC, C_max_, and plasma concentrations of doxepin and *N*-desmethyldoxepin in the genotype-based models are presented in Fig. 3, and detailed quantitative results are summarized in Table 4.

For doxepin, the fold error ranges were 1.00–1.27 for AUC and 0.77–1.35 for C_max_, while the corresponding GMFE values were 1.13 and 1.22, respectively. Overall, 63.3% and 85.7% of the doxepin plasma concentration fold errors were within the 1.25-fold and twofold error ranges, respectively.

For *N*-desmethyldoxepin, the fold error ranges were 1.11–2.35 for AUC and 0.74–1.15 for C_max_, and the GMFE values were 1.61 and 1.19, respectively. As genotype-specific plasma concentration data for *N*-desmethyldoxepin were not reported in the reference study, a direct comparison between the predicted and observed concentrations was not possible.

Overall, the PBPK model adequately predicted the pharmacokinetics of doxepin and *N*-desmethyldoxepin in both non-genotyped and genotype-specific evaluations, with predicted AUC and C_max_ values consistently within the predefined twofold acceptance criterion.

### Sensitivity analysis

**Fig. 4:**
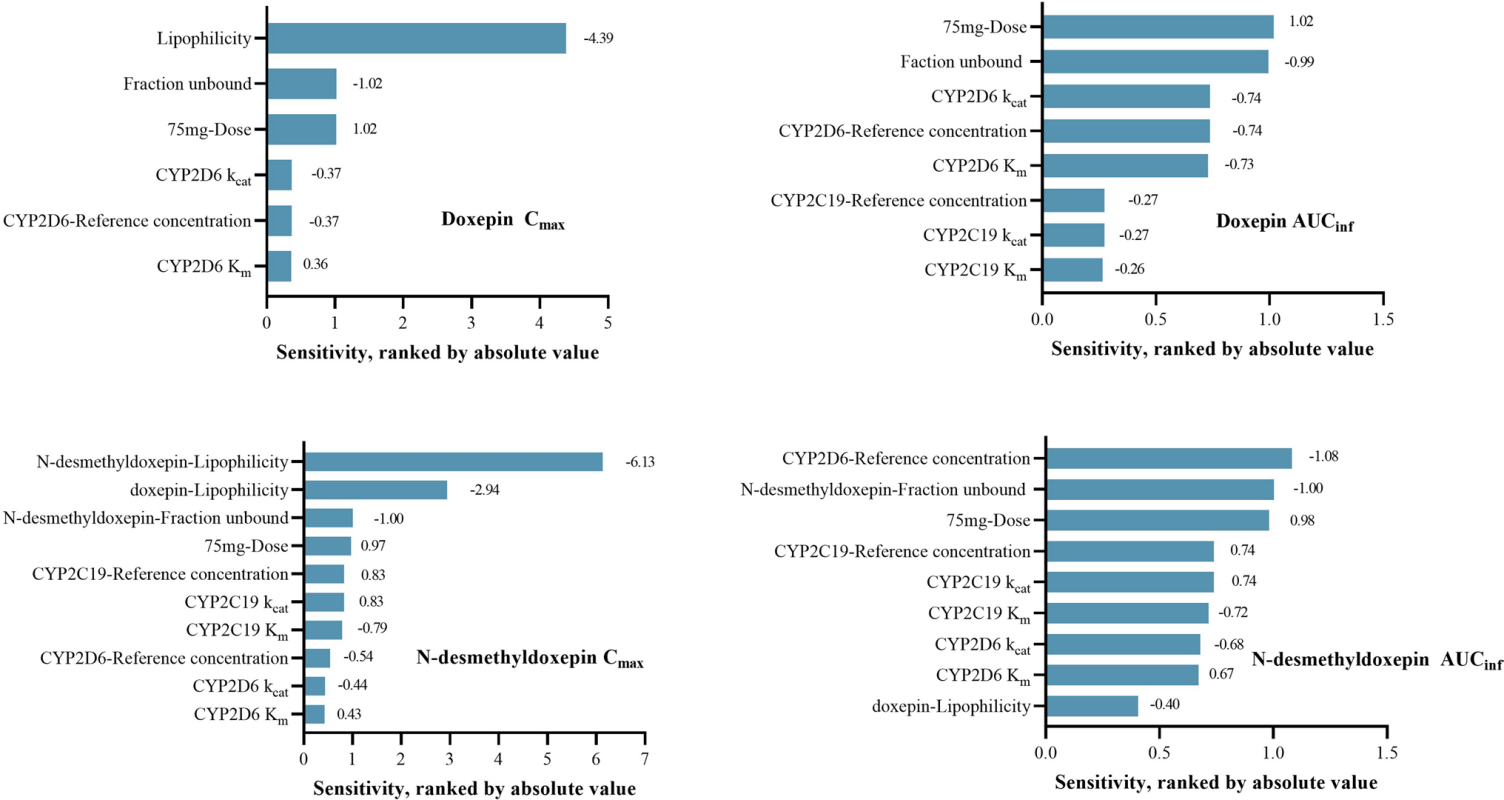
Results of sensitivity analysis of key input parameters affecting C_max_ and AUC_inf_ following a single oral doxepin (75 mg). Panels **A** and **B** show the sensitivity of doxepin C_max_ and AUC_inf_, respectively, whereas panels **C** and **D** show the sensitivity of *N*-desmethyldoxepin C_max_ and AUC_inf_, respectively. AUC_inf:_ area under the plasma concentration–time curve from 0 to infinity, C_max:_ maximum plasma concentration, K_m_: Michaelis–Menten constant, k_cat:_ turnover number

The results of the sensitivity analysis are presented in Fig. 4. Input parameters with sensitivity values greater than 0.5 were considered influential. The parameters identified as sensitive for AUC and C_max_ of doxepin and *N*-desmethyldoxepin are summarized below, listed in descending order of impact.

For doxepin, AUC was most sensitive to the administered dose, fraction unbound, CYP2D6 k_cat_, CYP2D6 reference concentration, and CYP2D6 K_m._ The C_max_ of doxepin was primarily influenced by lipophilicity, the fraction unbound, and the administered dose.

For *N*-desmethyldoxepin, the parameters most affecting AUC were the CYP2D6 reference concentration, fraction unbound, administered dose, CYP2C19 reference concentration, CYP2C19 k_cat_ and K_m_ (for doxepin metabolism), as well as the CYP2D6 k_cat_ and K_m_ values for doxepin. The C_max_ of *N*-desmethyldoxepin was sensitive to its own lipophilicity, the lipophilicity of doxepin, fraction unbound, administered dose, CYP2C19 reference concentration, CYP2C19 k_cat_ and K_m_ (for doxepin), and CYP2D6 reference concentration.

## Discussion

Several pharmacogenetic guidelines have recommended genotype-guided dosing of TCAs. The U.S. FDA Pharmacogenetic Associations Table suggests dose reduction in CYP2D6 PMs (FDA 2024), while the Clinical Pharmacogenetics Implementation Consortium (CPIC) and Dutch Pharmacogenetics Working Group (DPWG) recommend either dose reduction or alternative drugs for PM and IM phenotypes, and potential dose escalation in UMs (Hicks et al. 2017; Bousman et al. 2021, 2023; Brouwer et al. 2022; Beunk et al. 2024). Collectively, these recommendations underscore the clinical relevance of CYP2D6-mediated metabolism in doxepin therapy. However, discrepancies are observed regarding the type of pharmacogenomic guidance for the *CYP2C19*-doxepin pair (Kordou et al. 2021). In contrast, inconsistencies exist regarding pharmacogenetic guidance for the *CYP2C19*-doxepin gene-drug pair (Kordou et al. 2021). CPIC recommends consideration of alternative antidepressants in CYP2C19 PMs and UMs, reflecting concerns about altered doxepin exposure (Hicks et al. 2013). Conversely, the DPWG concluded that although CYP2C19 genetic variation influences doxepin plasma exposure, it does not significantly affect the combined exposure of doxepin and its active metabolite, *N*-desmethyldoxepin, which together are considered determinants of both therapeutic response and adverse effects. Accordingly, the DPWG does not recommend any clinical action based on CYP2C19 genotype for doxepin therapy (Kleine Schaars et al. 2026). Based on these considerations, the present study focused exclusively on CYP2D6 genotype–phenotype effects, in alignment with the DPWG guideline recommendations.

CYP2D6 is involved in the metabolism of approximately 20–25% of clinically used therapeutic drugs and is highly polymorphic, with more than 185 known alleles exhibiting a wide range of functional activities (https://www.pharmvar.org/gene/CYP2D6, accessed on Jan 10, 2026). For CYP2D6, an activity score is assigned to each allele, and an individual’s metabolic phenotype is classified based on the sum of the activity scores of the two alleles (genotype or diplotype) carried by that individual. Among representative alleles, *1, *2, *27, and *33 are assigned an activity score of 1.0; *17, *31, and *49 are assigned a score of 0.5; *10 is assigned a score of 0.25; and *3, *4, *5, *6, and *36 are assigned a score of 0 (https://www.clinpgx.org/page/cyp2d6RefMaterials, accessed on Jan 10, 2026). With respect to phenotype classification based on the sum of activity scores (*x*), the CPIC and DPWG previously applied different criteria. However, a consensus classification was published jointly by the two organizations in 2019, according to which UM are defined as *x* > 2.5, NM as 1.25 ≤ *x* ≤ 2.25, IM as 0 < *x* < 1.25, and PM as *x* = 0 (Caudle et al. 2020). In the clinical dataset used for model development in the present study (Kirchheiner et al. 2005), the activity scores of the study participants were uniformly 3.0 for UM, 2.0 for NM, 1.0 for IM, and 0 for PM.

In this study, PBPK models for doxepin and its active metabolite, *N*-desmethyldoxepin, were successfully developed and verified. The models adequately reproduced observed plasma concentration–time profiles, with most predicted AUC and C_max_ values for both compounds being within the twofold error criterion. These results indicate that the developed PBPK framework reliably captures the pharmacokinetic behavior of doxepin and *N*-desmethyldoxepin following oral administration.

The model's predictive performance was further supported by GMFE values close to unity for both doxepin and *N*-desmethyldoxepin. In addition to overall exposure metrics, visual predictive checks demonstrated good agreement between simulated and observed concentration–time profiles. Together, these findings confirm the suitability of the model for describing the systemic pharmacokinetics of doxepin and its active metabolite in a non-genotyped population.

The PBPK model was subsequently extended to evaluate the impact of *CYP2D6* genetic polymorphisms on doxepin pharmacokinetics. Simulations across CYP2D6 UM, NM, IM and PM phenotypes demonstrated a clear genotype-dependent trend in doxepin exposure, with increasing AUC and C_max_ observed as CYP2D6 metabolic capacity decreased. These trends were consistent with clinical observations and reflect the critical role of CYP2D6-mediated hydroxylation in doxepin clearance.

Similarly, *N*-desmethyldoxepin exposure exhibited an inverse relationship with CYP2D6 activity, reflecting differences in metabolic formation and elimination across phenotypes. Although genotype-specific plasma concentration–time profiles for *N*-desmethyldoxepin were not available in the reference clinical studies, the model adequately reproduced observed AUC and C_max_ values across CYP2D6 phenotypes. This limitation restricted model evaluation for the metabolite to exposure-based metrics. However, the consistency of predicted trends supports the mechanistic plausibility of the model.

Our PBPK model for doxepin was constructed based on its major metabolic pathways (PharmGKB 2024). The primary pathway included demethylation of doxepin to *N*-desmethyldoxepin, primarily mediated by CYP2C19. Minor contributions from CYP1A2, CYP3A4, and CYP2C9 were also incorporated as part of total hepatic clearance. In addition, both doxepin and *N*-desmethyldoxepin undergo hydroxylation primarily catalyzed by CYP2D6, which was explicitly integrated into the model to capture these reactions. This comprehensive approach allowed the model to reflect both major and minor metabolic routes of doxepin, aligning with established enzymatic pathways governing its metabolism.

Several limitations should be acknowledged. First, the PBPK model was developed using pharmacokinetic and pharmacogenomic data from healthy volunteers. Physiological differences between healthy subjects and patients with major depressive disorder, anxiety, or insomnia may influence drug metabolism and distribution. Second, although the *N*-desmethyldoxepin model was optimized using plasma concentration–time data, the absence of genotype-specific plasma data limited validation to exposure parameters (AUC and C_max_). Consequently, genotype-dependent profiles of the metabolite could not be fully assessed. Third, the model was based on single-dose data and may not capture long-term pharmacokinetic behavior. Chronic dosing could alter metabolism through enzyme induction or inhibition, affecting steady-state concentrations. Future work incorporating multiple-dose simulations may provide more clinically relevant insights into doxepin pharmacokinetics under chronic use.

As described above, individual activity scores may vary within each phenotype category. Because the present model was developed using fixed reference activity scores of 3.0 for UM, 2.0 for NM, 1.0 for IM, and 0 for PM, discrepancies between the model-recommended dosing and the optimal dose for a given individual may arise when that individual’s actual activity score differs from these reference values.

In conclusion, PBPK models for doxepin and *N*-desmethyldoxepin were successfully established and evaluated, incorporating *CYP2D6* genetic polymorphisms as a key source of interindividual variability. The models adequately predicted genotype-dependent differences in systemic exposure and provide a mechanistic framework for understanding the pharmacokinetics of doxepin and its active metabolite. This PBPK framework can serve as a valuable tool to predict doxepin pharmacokinetics under various clinical conditions, considering genetic variability and interindividual differences in metabolic capacity. The findings of this study may contribute to optimizing personalized dosing strategies for doxepin therapy.

## Data Availability

The datasets used and/or analyzed during the current study are available from the corresponding author upon reasonable request.
